# Testosterone therapy in patients with heart failure and protein-calorie malnutrition: Insights from a propensity-matched cohort study

**DOI:** 10.1016/j.cpcardiol.2025.103070

**Published:** 2025-04-29

**Authors:** SilvioNunes Augusto, David C. Kaelber, W.H. Wilson Tang

**Affiliations:** a Cardiovascular and Metabolic Sciences, Lerner Research Institute, Cleveland OH, USA; b The Center for Clinical Informatics Research and Education, The MetroHealth System, Cleveland OH, USA; c The Departments of Internal Medicine, Pediatrics, and Population and Quantitative Health Sciences, Case Western Reserve University, Cleveland OH, USA; d Heart, Vascular and Thoracic Institute, Cleveland Clinic, Cleveland OH, USA; e Cleveland Clinic Lerner College of Medicine of Case Western Reserve University, Cleveland OH, USA

**Keywords:** Heart failure, Prognosis, Testosterone therapy

## Abstract

**Introduction:**

Testosterone therapy may improve physical capacity and mood in men with chronic heart failure. However, recent findings have raised concerns that testosterone therapy could also increase the risk of atrial fibrillation, pulmonary embolism, and acute kidney injury.

**Methods:**

Leveraging data from the TriNetX platform from January 1, 2014, to December 01, 2024, we conducted a propensity-matched analysis of two cohorts of patients with heart failure with protein-calorie malnutrition, where the only difference between cohorts was testosterone therapy. The primary outcomes were all-cause mortality and the incidence of acute heart failure, with secondary outcomes including major adverse cardiovascular events as well as cardiovascular, kidney, and thrombotic diseases.

**Results:**

After propensity matching, 577 patients were compared across the two cohorts. The findings indicated that testosterone therapy reduced the risk of all-cause mortality (hazard ratio [HR] 0.56, 95 % confidence interval [CI] 0.46–0.68), acute heart failure (HR 0.62, 95 % CI 0.49–0.78), and major adverse cardiovascular events (HR 0.77, 95 % CI 0.61–0.97). Additionally, incident peripheral arterial disease (HR 0.52, 95 % CI 0.30–0.91), coronary arterial disease (HR 0.83, 95 % CI 0.69–1.00), acute myocardial infarction (HR 0.65, 95 % CI 0.48–0.89), and atrial fibrillation (HR 0.79, 95 % CI 0.66–0.95) also favored the testosterone-treated cohort. There was a trend toward an increased risk of venous thromboembolism (HR 1.14, 95 % CI 1.03–1.27) and stroke (HR 1.12, 95 % CI 0.81–1.54) associated with testosterone therapy.

**Conclusion:**

These hypothesis-generating findings support potential beneficial effects of testosterone therapy in patients with heart failure and protein-calorie malnutrition.

## Introduction

Heart failure is often associated with muscle wasting and metabolic shifts favoring catabolism, reducing muscle mass and strength. Testosterone replacement therapy can enhance exercise capacity and muscle strength, improve insulin sensitivity and glucose metabolism, and positively affect baroreflex sensitivity and cardiac output.^1–5^ However, concerns have been raised regarding cardiovascular risk of testosterone use,^6^ which may induce platelet aggregation by stimulating thromboxane A2 that may contribute to increased thrombogenicity.^7^ In fact, a systematic review of 45 trials found that testosterone supplementation may increase cardiovascular events in patients who are 65 years or above.^8^ Importantly, a cardiovascular safety trial, the TRAVERSE (Testosterone Replacement Therapy for Assessment of Long-term Vascular Events and Efficacy Response in Hypogonadal Men) study found that in men with hypogonadism and preexisting or a high risk of cardiovascular disease, testosterone-replacement therapy was noninferior to placebo with respect to the incidence of major adverse cardiac events.^9^

Protein-calorie malnutrition in patients with heart failure has been linked to higher mortality rates and increased morbidity. Studies have shown that malnourished patients have a higher risk of in-hospital mortality, longer hospital stays, and increased rates of complications such as cardiac arrest and cardiogenic shock.^10–12^ In this observational retrospective cohort study using the TriNetX platform, we sought to investigate whether patients with heart failure and protein-calorie malnutrition would uniquely benefit from testosterone therapy.

## Methods

### Data Availability.

The data, analytic methods, and study materials were collected from the TriNetX database, which includes deidentified electronic medical records from 102 healthcare organizations (HCOs). This study utilized data from January 1, 2014, to December 01, 2024. Institutional Review Board (IRB) approval was not required as the study involved a secondary analysis of deidentified data, exempt from informed consent per the HIPAA Privacy Rule Section §164.514(a). The de-identification process was certified by a qualified expert according to Section §164.514(b)(1), with the most recent certification completed in December 2020. This observational retrospective cohort study followed the STROBE guidelines.^13^

### Study population.

The study period spanned from January 1, 2014, to December 01, 2024, and included men older than 18 years, with a follow-up period of 5 years post-index date. The exclusion criteria for both cohorts included a diagnosis of heart failure prior to January 1, 2014. The inclusion criteria included patients with at least one encounter diagnosis of heart failure (ICD-10-CM: I.50) and a diagnosis of protein-calorie malnutrition (ICD-10-CM: E44, E44.0, E44.1, E46, E43). The treatment cohort included individuals prescribed testosterone (RxCUI: 10379), while the non-treatment cohort had no testosterone prescriptions during the examined time periods. A minimum of two instances of testosterone therapy was required to exclude patients who had only a single instance. The time window for the baseline characteristics table was established based on the closest values within 1 year prior to the index event. The most recent occurrence data were collected to ensure comparability between the cohorts, and propensity score matching was performed based on the following variables: In the demographics category, patients were matched on age, sex, race, and ethnicity. In the diagnosis category, patients were matched on hypertension (%), hyperlipidemia (%), and atrial fibrillation and flutter (%). In the medication category, patients were matched on beta-blocking agents (%), calcium channel blockers (%), angiotensin II receptor blockers (ARBs) (%), diuretics (%), and ACE inhibitors (%). In the laboratory category, patients were matched on BMI (kg/m^2^), body weight (kg), left ventricular ejection fraction (LVEF) (%), triglycerides (mg/dL), cholesterol (mg/dL), HDL cholesterol (mg/dL), LDL cholesterol (mg/dL), glucose (mg/dL), hemoglobin (g/dL), bicarbonate (mmol/L), iron (μg/dL), urea nitrogen (mg/dL), creatinine (mg/dL), estimated glomerular filtration rate (eGFR) (mL/min/1.73m^2^), natriuretic peptide B (pg/mL), and C-reactive protein (mg/L). (See Supplemental File)

### Study endpoints & statistical analysis.

The study outcomes included all-cause mortality, and the occurrence of encounter diagnoses for acute heart failure (ICD-10-CM: I50.21, I50.23, I50.31, I50.33, I50.41, I50.43, I50.811, I50.813), major adverse cardiovascular events (ICD-10-CM: I21, I21.2, I21.3, I21.4, I61, I63, I69), stroke (ICD-10-CM: I63, I67.81, I67.2), peripheral arterial disease (ICD-10-CM: I70.2, I70.20, I70.22, I70.201, I70.202, I70.29), coronary arterial disease (ICD-10-CM: I25.10, I25.110), acute myocardial infarction (ICD-10-CM: I21, I21.9), atrial fibrillation (ICD-10-CM: I48, I48.9, I48.91, I48.0, I48.1, I48.2, I48.20), venous thromboembolism (ICD-10-CM: I82, I82.9, I26, I26.9, I26.99), and chronic kidney disease (ICD-10-CM: N18, N18.1, N18.2, N18.3, N18.4, N18.5). Risk differences, risk ratios, and odds ratios were calculated for each outcome with accompanying 95 % confidence intervals. Kaplan-Meier survival analyses were conducted to estimate survival probabilities, and hazard ratios were derived using Cox proportional hazards models. Baseline balance was assessed using standardized mean differences, with values below 0.2 considered indicative of adequate matching.

## Results

### Baseline characteristics.

**Fig. 1.**
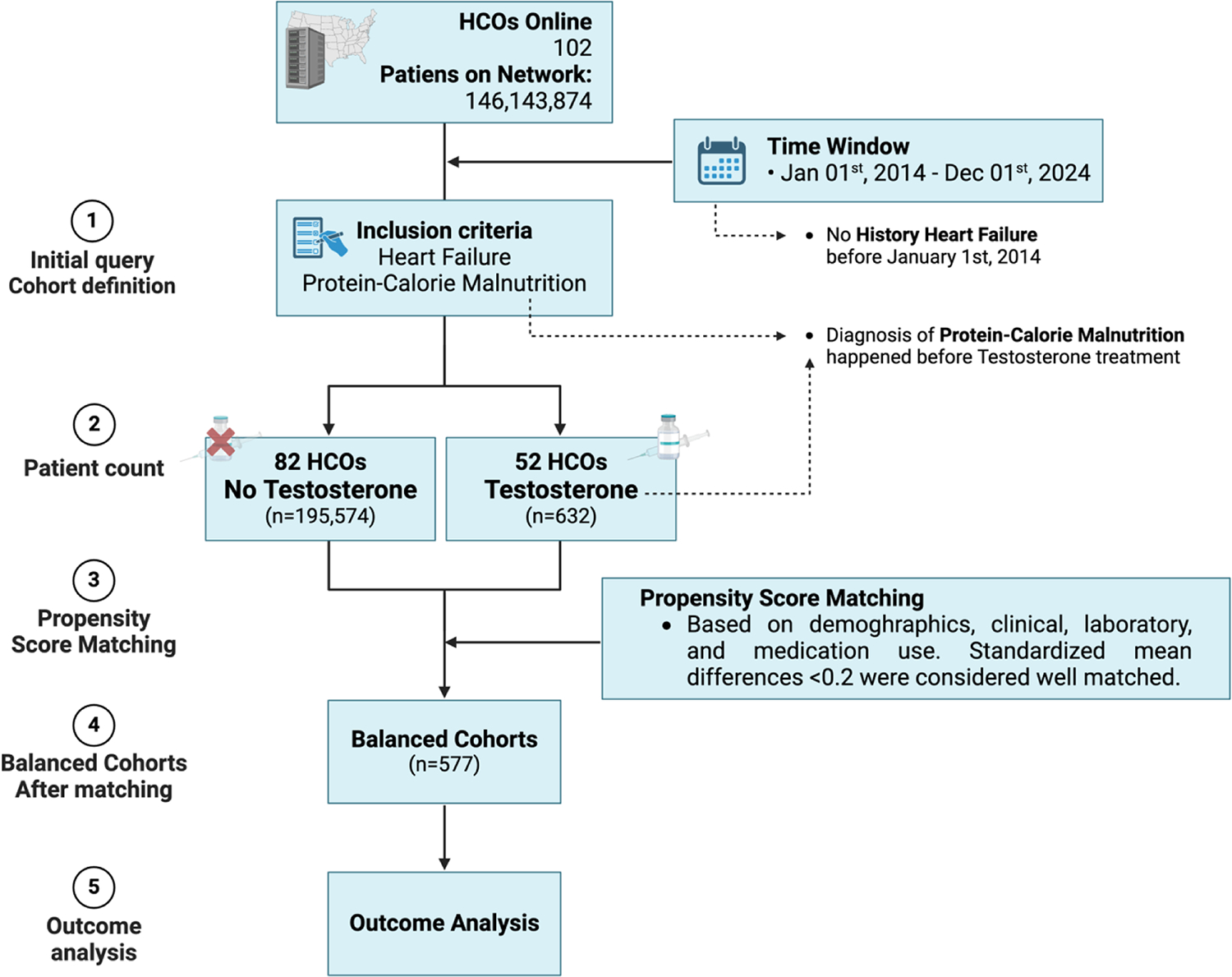
STROBE Diagram. The inclusion criteria for patients included a diagnosis of heart failure (I50) and protein-calorie malnutrition (E43, E44, E44.0, E44.1, E46). After propensity score matching based on demographics, clinical, laboratory results, and medication usage, both cohorts comprised 577 patients each. Standardized mean differences of <0.2 were deemed well-matched. Patients had no prior history of heart failure before January 1st, 2014. Testosterone treatment was initiated only after the diagnosis of protein-calorie malnutrition.

Fig. 1 shows the flow chart of patient selection and describes propensity score matching variables up to outcome analysis. Before propensity score matching, the non-treatment cohort consisted of 195,574 patients, while the treatment cohort included 632 patients. Following matching, each cohort included 577 patients, achieving balance across all baseline variables with standardized mean differences all <0.2, with exception of creatinine (Table 1).

### Clinical outcomes.

**Fig. 2.**
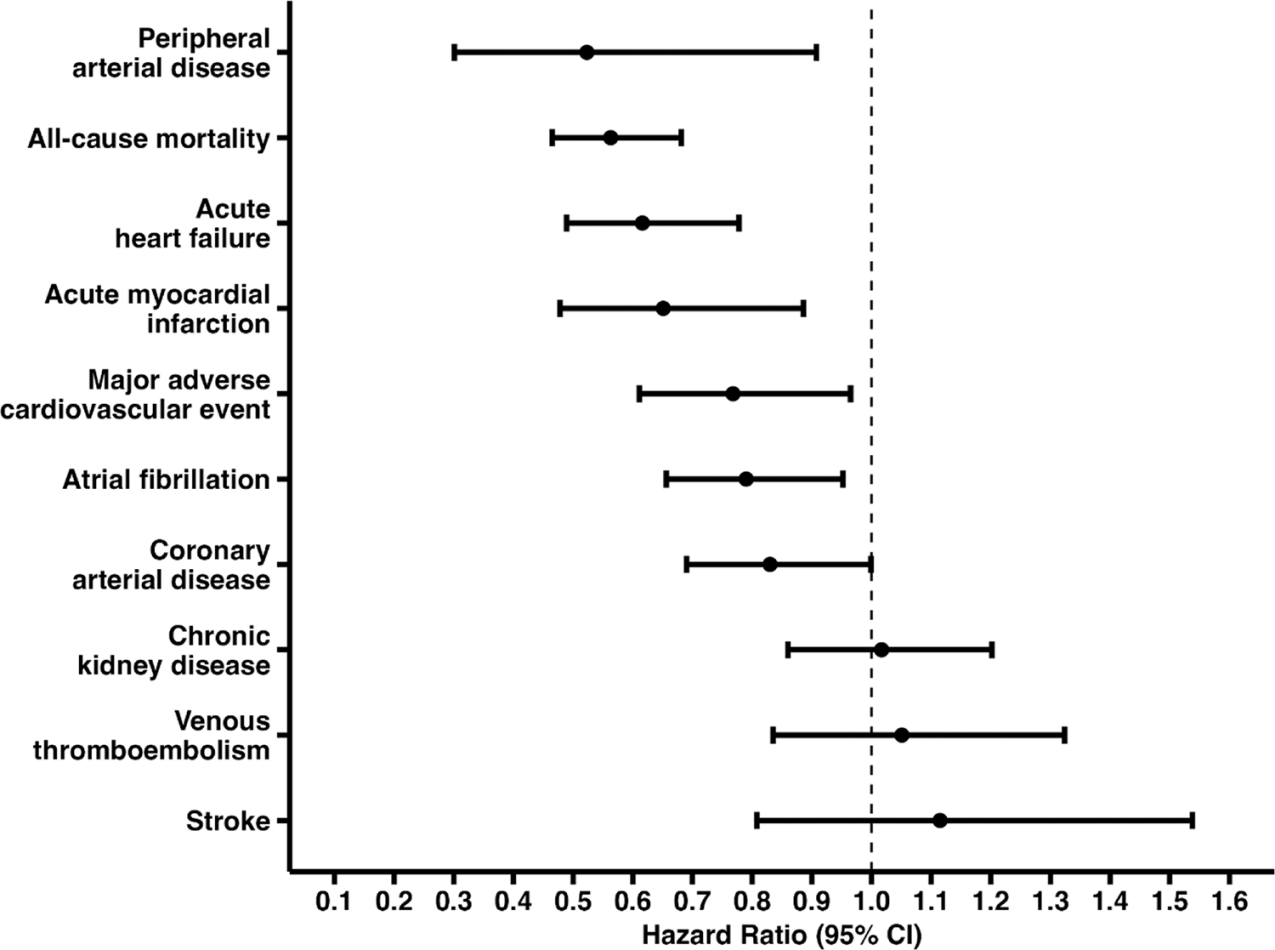
Forest Plot Hazard-Ratio for main cohort analysis. Fig. 2A shows all outcomes of the main cohort analysis. The results indicated a significantly reduced risk for peripheral arterial disease, all-cause mortality, acute heart failure, acute myocardial infarction, major adverse cardiovascular events, atrial fibrillation, and coronary arterial disease, all favoring testosterone treatment. Although not statistically significant, chronic kidney disease, stroke, and venous thromboembolism show a trend towards increased risk.

**Fig. 3.**
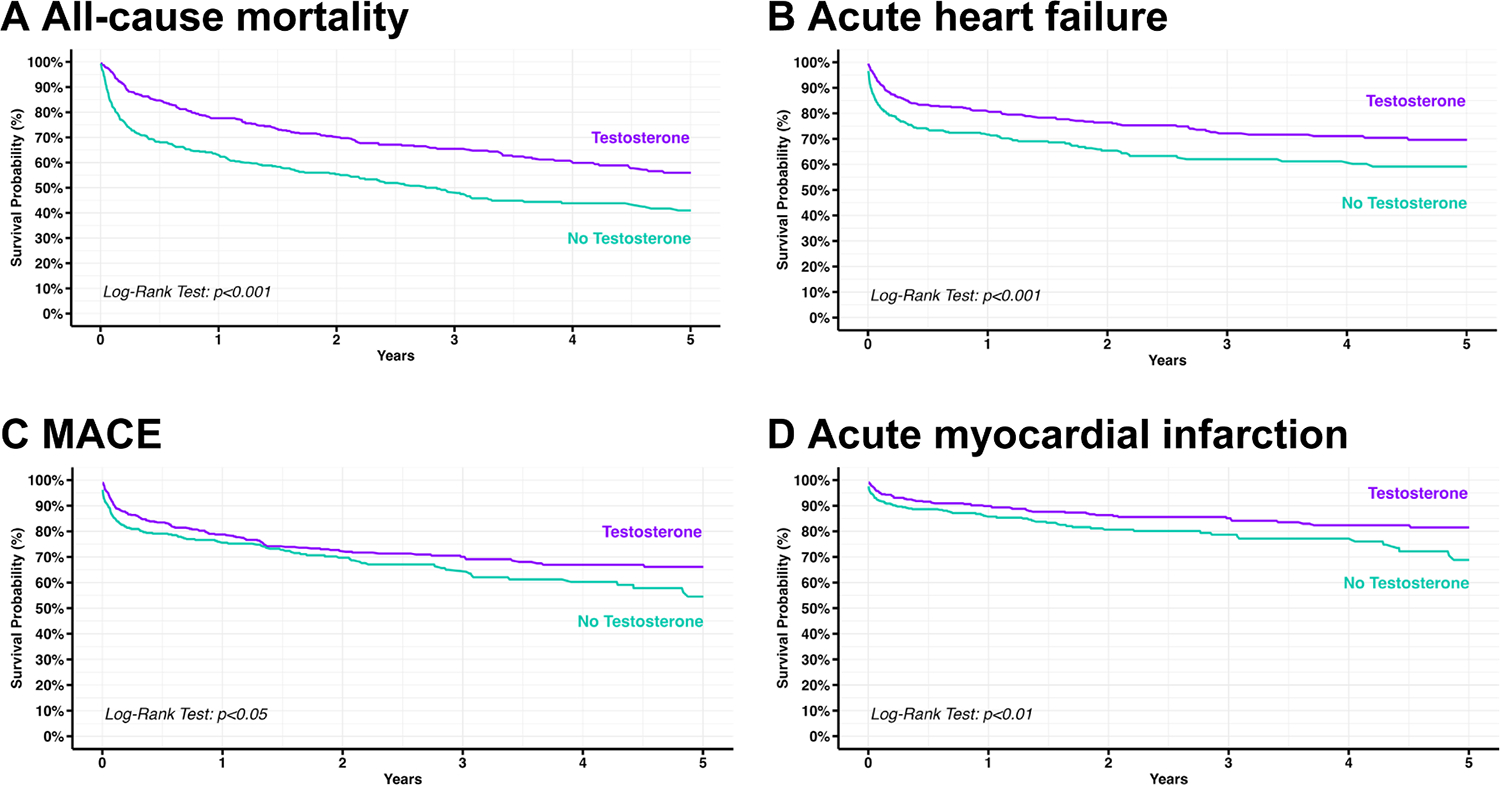
Kaplan-Meier curves for all-cause mortality, acute heart failure, major adverse cardiovascular events, and acute myocardial infarction. Fig.s 3A–3D shows the Kaplan-Meier curve for A) all-cause mortality (log-rank test *p* < 0.001), B) acute heart failure (log-rank test *p* < 0.05), and C) major adverse cardiovascular events (log-rank test *p* < 0.001), and D) acute myocardial infarction (log-rank test *p* < 0.01).

Hazard ratios, risk differences, risk-ratios, and odds-ratios for the main cohort analysis are available in Table 2 and Fig. 2. At the end of the five-year follow-up, patients in the testosterone therapy cohort experienced reduced the risk of all-cause mortality (hazard ratio [HR] 0.56, 95 % confidence interval [CI] 0.46–0.68, Fig. 3A), acute heart failure (HR 0.62, 95 % CI 0.49–0.78, Fig. 3B), major adverse cardiovascular events (HR 0.77, 95 % CI 0.61–0.97, Fig. 3C), and acute myocardial infarction (HR 0.65, 95 % CI 0.48–0.89, Fig. 3D), but not for chronic kidney disease (HR 1.02, 95 % CI 0.86–1.20). Additionally, incident peripheral arterial disease (HR 0.52, 95 % CI 0.30–0.91), coronary arterial disease (HR 0.83, 95 % CI 0.69–1.00), and atrial fibrillation (HR 0.79, 95 % CI 0.66–0.95) also favored the testosterone-treated cohort. There was a trend toward an increased risk of venous thromboembolism (HR 1.14, 95 % CI 1.03–1.27) and stroke (HR 1.12, 95 % CI 0.81–1.54) associated with testosterone therapy, but did not reach statistical significance.

## Discussion

Using a real-world cohort of patients with heart failure and protein-calorie malnutrition, we observed that testosterone therapy significantly reduced adverse clinical outcomes and incident development of cardio-renal disease despite an increased risk for venous thrombosis. Because heart failure is commonly associated with muscle wasting, our findings provide a compelling argument for conducting future studies to investigate the potential benefits of testosterone replacement therapy in men with heart failure and coexisting protein-calorie malnutrition.

Based on a real-world cohort, our findings indicate significant reductions in the risks of adverse clinical outcomes in patients with heart failure and documented protein-calorie malnutrition. Considering the most recent EMAS statement from 2023,^14^ as well as the concerns raised by the TRAVERSE trial, regarding increased risk for acute kidney injury, pulmonary embolism, and atrial fibrillation with testosterone treatment in a largely non-heart failure cohort,^9^ our findings are reassuring that testosterone replacement therapy may be a viable option for a specific cohort of heart failure patients suffering from protein calorie malnutrition. Various small studies have demonstrated that testosterone administration improves maximal exercise capacity, as measured by peak oxygen consumption (VO_2_), and increases muscle strength in patients with heart failure.^15^ In elderly patients with chronic heart failure, long-acting testosterone therapy has been associated with improvements in exercise capacity and muscle strength, thereby relieving heart failure symptoms.^1,2^ Testosterone has also been shown to improve insulin sensitivity and glucose metabolism, which can help better utilize nutrients, which is crucial for patients suffering from malnutrition.^1,3,16–18^ Additionally, testosterone therapy was associated with improvements in baroreflex sensitivity and cardiac output, as well as 6-minute walk distances and diastolic function.^1,4,5^ However, there were also conflicting studies that failed to demonstrate significant improvements in functional capacity, clinical symptoms, or cardiac function despite a substantial rise in serum testosterone levels with testosterone treatment.^19^ Despite improvement in body composition (both fat and lean mass) and upper body strength in some studies, functional performance or aerobic capacity or strength did not improve without exercise.^20,21^

The risk of experiencing thromboembolic events with testosterone therapy has been well documented.^22–24^ For instance, a population-based case-control study found that the risk of venous thromboembolism was elevated during the first six months of testosterone treatment but not after more than six months of treatment.^23^ Additionally, a case-crossover study published in JAMA Internal Medicine found that testosterone therapy was associated with a higher risk of venous thromboembolism in men with and without hypogonadism.^24^ This temporal association highlights the importance of monitoring patients closely, especially during the initial phase of testosterone therapy.^22^ It is important to note that testosterone therapy is contraindicated in those with recent cardiovascular events or those actively trying to conceive. Despite not being statistically significant, the increased venous thromboembolism and stroke incidence observed in our cohort was expected and thus served as a confirmation of the validity of our propensity-matching. Previous studies have found that testosterone replacement therapy is likely beneficial for patients with chronic kidney disease.^25,26^ However, testosterone supplementation has been associated with increased blood pressure, sodium and water retention, and activation of vasoconstrictor systems in the kidney, such as the renin-angiotensin system and endothelin, which can compromise renal function.^27^ Given our results, careful monitoring and consideration of the patient’s renal status are essential when initiating testosterone therapy in heart failure patients. While testosterone therapy does not directly cause CKD, it can exacerbate renal dysfunction in patients with pre-existing kidney disease.

Some limitations faced during this research are related to our data source. This study used propensity score matching to reduce bias between cohorts and leverage a large and diverse cohort from the TriNetX platform. Our sample size is dependent on data availability from the HCO’s part of the TriNetX Research Network. Moreover, some inherent limitations of retrospective analyses remain, as it is not possible to fully eliminate residual or unmeasured confounding inherent in observational data. Since the study’s design depends on pre-existing records not initially collected for research purposes, the accuracy and completeness of the information cannot be guaranteed. The outcome of all-cause mortality should also be interpreted carefully, as a death event outside the hospital setting might not be appropriately accounted for in the system until social security number verification – it is important to emphasize we do not have direct access to patient information. Also, with respect to testosterone replacement therapy, all we could look for was the presence of two or more testosterone prescriptions. We could not assess patient compliance with the medication or any dose response. However, these limitations in understanding the testosterone used in the testosterone group probably mean that, if anything, because many patients do not take medications as directed, our findings under-represent the true effect of testosterone in patients taking it regularly. Further studies are warranted to test the feasibility and clinical benefits of testosterone therapy in this highly vulnerable patient population.

## Conclusion

Our hypothesis-generating findings support the hypothesis that testosterone therapy can significantly reduce adverse cardiovascular events and the development of cardiovascular diseases in patients with heart failure and protein-calorie malnutrition.

## Supplementary Material

Supplemental material

## Figures and Tables

**Table 1:** Baseline characteristics between cohorts before and after propensity matching.

	Before Propensity Score Matching				After Propensity Score Matching			
	Cohort 1 (Mean ± SD)	Cohort 2 (Mean ± SD)	P-Value	Std diff.	Cohort 1 (Mean ± SD)	Cohort 2 (Mean ± SD)	P-Value	Std diff.
**Demographics**								
Current Age	64.9 ± 14.8	73.6 ± 13.6	<0.001	0.612	65.1 ± 14.6	65.5 ± 15.8	0.67	0.025
Black or African American	13 %	19 %	<0.001	0.187	13 %	15 %	0.233	0.07
White	74 %	65 %	<0.001	0.196	74 %	73 %	0.74	0.02
**Clinical**								
Left Ventricular Ejection Fraction (LVEF) (%)	55.2 ± 13.8	49.7 ± 15.9	0.018	0.367	55.2 ± 13.8	53.1 ± 14.7	0.45	0.148
BMI (kg/m2)	27.1 ± 7.1	25.8 ± 6.8	<0.001	0.196	27.2 ± 7.1	26.2 ± 7.1	0.057	0.129
Body Weight (lbs)	186.9 ± 53.1	176.4 ± 50.3	<0.001	0.204	187.2 ± 53.1	181.6 ± 52.8	0.128	0.107
Hyperlipidemia (%)	47 %	38 %	<0.001	0.169	47 %	50 %	0.317	0.059
Hypertension (%)	66 %	53 %	<0.001	0.263	66 %	67 %	0.662	0.026
A-fib (%)	42 %	30 %	<0.001	0.256	41 %	45 %	0.235	0.07
**Laboratory**								
Total Cholesterol (mg/dL)	143.7 ± 62.8	137.4 ± 46.9	0.022	0.114	143.4 ± 63.1	145.2 ± 66.2	0.745	0.027
LDL Cholesterol (mg/dL)	75.4 ± 43.1	72.8 ± 36.8	0.233	0.065	75.3 ± 43.2	76.8 ± 43.2	0.683	0.035
Triglycerides (mg/dL)	163.5 ± 235.9	120.1 ± 99.1	<0.001	0.24	163.2 ± 237.7	149.2 ± 107.3	0.338	0.076
Glucose (mg/dL)	122.8 ± 53.7	122.0 ± 53.1	0.726	0.015	123.1 ± 53.9	122.8 ± 53.8	0.914	0.007
Hemoglobin (g/dL)	10.6 ± 2.7	10.9 ± 2.4	0.001	0.142	10.6 ± 2.7	10.4 ± 2.6	0.373	0.056
BNP (pg/mL)	775.7 ± 1682.0	1065.9 ± 3169.4	0.256	0.114	784.7 ± 1691.2	909.7 ± 2451.2	0.601	0.059
NT-proBNP (pg/mL)	5408.1 ± 8940.2	6567.8 ± 11062.3	0.268	0.115	5502.4 ± 9037.0	7407.9 ± 12146.0	0.163	0.178
C Reactive Protein (mg/L)	56.0 ± 70.0	64.7 ± 74.7	0.094	0.119	55.0 ± 69.4	57.9 ± 71.9	0.677	0.041
Bicarbonate (mmol/L)	25.1 ± 4.6	25.5 ± 4.5	0.05	0.084	25.1 ± 4.6	25.2 ± 4.7	0.753	0.019
Iron (μg/dL)	59.8 ± 42.2	54.2 ± 44.2	0.034	0.131	60.0 ± 42.3	53.1 ± 38.0	0.045	0.172
Urea Nitrogen (mg/dL)	32.9 ± 21.5	28.5 ± 20.4	<0.001	0.209	32.8 ± 21.6	32.0 ± 22.2	0.537	0.039
Creatinine (mg/dL)	1.7 ± 1.6	1.7 ± 2.0	0.423	0.039	1.7 ± 1.6	2.1 ± 2.3	<0.001	0.229
GFR (CKD-EPI) (mL/min/1.73m2)	67.3 ± 45.7	67.6 ± 41.1	0.873	0.007	67.1 ± 45.6	64.2 ± 44.2	0.301	0.064
**Medications**								
Beta Blockers (%)	62 %	48 %	<0.001	0.281	62 %	65 %	0.299	0.061
Calcium Channel Blockers (%)	41 %	27 %	<0.001	0.295	40 %	45 %	0.137	0.088
Angiotensin II Inhibitors (%)	17 %	15 %	0.077	0.071	17 %	17 %	0.938	0.005
Diuretics (%)	70 %	46 %	<0.001	0.501	70 %	71 %	0.699	0.023
ACE Inhibitors (%)	19 %	19 %	0.975	0.001	19 %	19 %	0.94	0.004

Numerical data is expressed in mean ± standard deviation, and categorical data as count (%). Abbreviations: Std diff, standard difference; ACE, Angiotensin-converting enzyme; BMI, body mass index; LDL, low-density lipoprotein; HDL, high-density lipoprotein, BNP, B-type natriuretic peptide; GFR, glomerular filtration rate; ACE, angiotensin converting enzyme.

**Table 2:** Risk difference, risk ratio, and odds ratio for primary and secondary outcomes.

	Hazard Ratio (HR)	Kaplan-Meier log-rank test			Risk Difference (RD)	Risk Ratio (RR)	Odds Ratio (OR)
Outcomes	HR (95 %CI)	X^2^	df	p-value	RD (95 %CI)	RR (95 %CI)	OR (95 %CI)
All-cause mortality	0.56 (0.47, 0.68)	35.98	1	< 0.001	− 12.41 % (− 18.02 %,− 6.80 %)	0.72 (0.62, 0.84)	0.59 (0.46, 0.75)
Major adverse cardiovascular event	0.77 (0.61, 0.96)	5.14	1	0.023	− 1.04 % (− 6.08 %,4.00 %)	0.96 (0.79, 1.17)	0.95 (0.73, 1.23)
Acute heart failure	0.62 (0.49, 0.78)	16.99	1	< 0.001	− 6.07 % (− 11.06 %,− 1.07 %)	0.78 (0.64, 0.96)	0.72 (0.55, 0.95)
Coronary arterial disease	0.83 (0.69, 1.00)	3.86	1	0.0496	1.04 % (− 4.59 %,6.67 %)	1.03 (0.89, 1.19)	1.04 (0.82, 1.32)
Peripheral arterial disease	0.52 (0.30, 0.91)	5.49	1	0.019	− 1.91 % (− 4.32 %,0.51 %)	0.66 (0.38, 1.12)	0.64 (0.37, 1.13)
Stroke	1.11 (0.81, 1.54)	0.44	1	0.508	3.81 % (− 0.08 %,7.71 %)	1.34 (0.99, 1.81)	1.4 (0.99, 1.97)
Acute myocardial infarction	0.65 (0.48, 0.89)	7.54	1	0.006	− 3.12 % (− 7.14 %,0.91 %)	0.8 (0.60, 1.07)	0.77 (0.56, 1.08)
Atrial fibrillation	0.79 (0.66, 0.95)	6.11	1	0.013	− 0.52 % (− 6.14 %,5.10 %)	0.99 (0.85, 1.14)	0.98 (0.77, 1.24)
Chronic kidney disease	1.02 (0.86, 1.20)	0.05	1	0.827	8.84 % (3.10 %,14.58 %)	1.2 (1.06, 1.36)	1.43 (1.13, 1.80)
Venous thromboembolism	1.05 (0.84, 1.32)	0.18	1	0.668	4.85 % (− 0.16 %,9.86 %)	1.21 (0.99, 1.48)	1.29 (0.99, 1.69)

Abbreviations: CI, confidence interval; df, degrees of freedom.
