# Supplementary material for: Testosterone therapy in patients with heart failure and protein-calorie malnutrition: Insights from a propensity-matched cohort study
Source: Curr Probl Cardiol. Author manuscript; Available in PMC 2025 Jul 1. (PMC12129651; doi:10.1016/j.cpcardiol.2025.103070)
Supplement: Supplemental material [file NIHMS2081394-supplement-Supplemental_material.docx]

# **Supplemental File 1. Propensity Score Matching**

The system generates a propensity score for each patient in each cohort. The propensity score ranges between 0 and 1 and indicates the predicted probability a patient is in cohort B, given the patient’s covariates. TriNetX uses logistic regression to generate the propensity scores by implementing the well-tested, standard software package [scikit-learn](https://scikit-learn.org/). Specific code and methods can be verified at <https://live.trinetx.com/>. Propensity score matching was performed on 37 characteristic(s). In the demographics category, patients were matched on age, sex, race, and ethnicity. In the diagnosis category, patients were matched on hypertension (%), hyperlipidemia (%), and atrial fibrillation and flutter (%). In the medication category, patients were matched on beta-blocking agents (%), calcium channel blockers (%), angiotensin II receptor blockers (ARBs) (%), diuretics (%), and ACE inhibitors (%). In the laboratory category, patients were matched on BMI (kg/m²), body weight (kg), left ventricular ejection fraction (LVEF) (%), triglycerides (mg/dL), cholesterol (mg/dL), HDL cholesterol (mg/dL), LDL cholesterol (mg/dL), glucose (mg/dL), hemoglobin (g/dL), bicarbonate (mmol/L), iron (μg/dL), urea nitrogen (mg/dL), creatinine (mg/dL), estimated glomerular filtration rate (eGFR) (mL/min/1.73m²), natriuretic peptide B (pg/mL), and C-reactive protein (mg/L). Characteristics of the cohorts before and after matching are summarized in the table below.

**Testosterone cohort:** This query was run on the network Research with 103 HCO(s) queried and 103 HCO(s) responded. A total of 52 provider(s) responded with patients. The final cohort included 632 patients who matched the query criteria.

**No Testosterone cohort:** This query was run on the network Research with 102 HCO(s) queried and 102 HCO(s) responded. A total of 82 provider(s) responded with patients. The final cohort included 195,574 patients who matched the query criteria.

| **Cohort 1 and cohort 2 patient count before and after propensity score matching** | | | |
| --- | --- | --- | --- |
| Cohort | Patient count before matching | | Patient count after matching |
| 1 - Testosterone | 583* | | 577 |
| 2 – No Testosterone | 170,974* | | 577 |
| **Propensity score density function - Before and after matching (cohort 1 - purple, cohort 2 - green)** | | | |
| 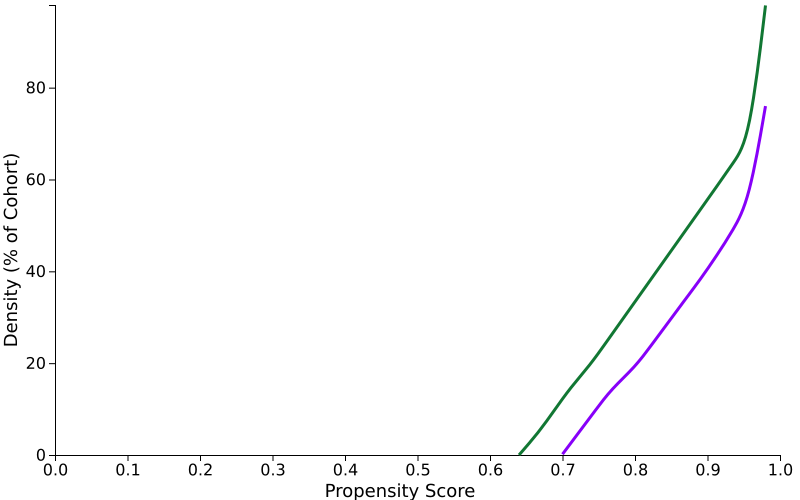 | | 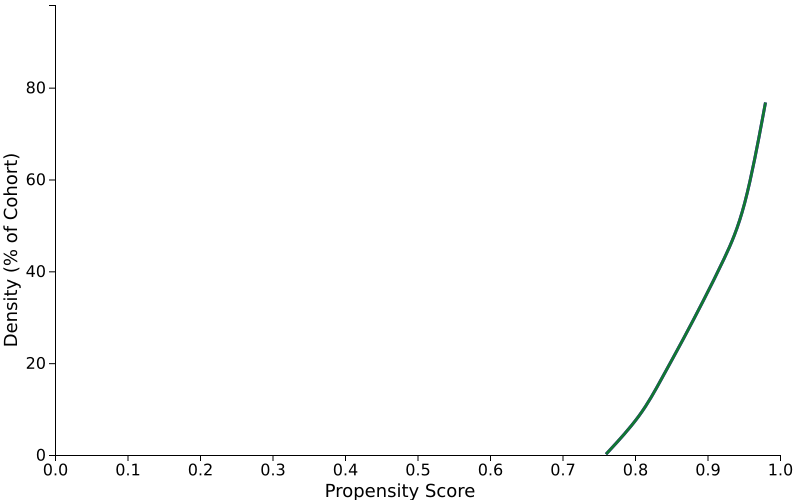 | |

* TriNetX numbers represent the exact patient count available at the time of the analysis. Data availability is dependent on the TriNetx platform.
